# Effects of Exercise Intensity and Duration on Acute-Phase Proteins in Thoroughbred Racehorses

**DOI:** 10.3390/ani16060977

**Published:** 2026-03-20

**Authors:** Chiara Storoni, Blagoje Dimitrijević, Gabriel Otava, Yubao Li, Fulvio Laus, Vincenzo Cuteri

**Affiliations:** 1 School of Biosciences and Veterinary Medicine, University of Camerino, Matelica, via Circonvallazione 93/95, 62024 Camerino, Italy; chiara.storoni@unicam.it; 2 Freelance Veterinarian, 11000 Beograd, Serbia; blagoje.vet@gmail.com; 3 Clinic for Reproduction, Faculty of Veterinary Medicine Timisoara, University of Life Sciences “King Michael I”, 300645 Timisoara, Romania; gabiotava@yahoo.com; 4 School of Pharmaceutical Sciences and Food Engineering, Liaocheng University, Liaocheng 252000, China; liyubao@lcu.edu.cn

**Keywords:** exercise physiology, acute-phase response, serum amyloid A, equine, inflammation, endurance exercise, gallop race

## Abstract

Physical exercise represents a physiological stressor capable of activating the acute-phase response (APR) in horses. However, the relative contribution of exercise intensity *versus* duration to acute-phase protein (APP) dynamics remains incompletely defined. This study compared the effects of short, high-intensity gallop exercise (2400 m flat race; *n* = 12) and prolonged, low-intensity endurance exercise (40 km; *n* = 13) on serum amyloid A (SAA), haptoglobin (Hp), and ceruloplasmin (Cp) in Thoroughbred racehorses. Blood samples were collected before exercise and at defined post-exercise time points. Between-group comparisons were performed at shared time points (72 h and 96 h post-exercise) using mixed-effects modeling and effect size analysis. The significant Group × Time interaction for SAA indicates that exercise duration plays a key role in determining the magnitude and persistence of the late-phase systemic inflammatory response. Serum amyloid A emerged as the most sensitive biomarker of cumulative physiological stress following prolonged exercise. These findings support the use of SAA monitoring during recovery to assist training management in equine athletes. Because early post-exercise sampling was not performed in the gallop group, conclusions primarily reflect differences in late-phase (72–96 h) APP kinetics.

## Simple Summary

Physical exercise can induce a transient inflammatory response in horses, which may reflect physiological adaptation or, if excessive, impaired recovery. Monitoring acute-phase proteins (APPs) may help distinguish these conditions and support training management. In this study, we compared the effects of short, high-intensity exercise (gallop racing) and prolonged, low-intensity exercise (endurance racing) on selected APPs in racehorses. Our results show that endurance exercise induces a more pronounced and prolonged late-phase systemic response than gallop exercise, and they highlight serum amyloid A (SAA) as a sensitive biomarker of exercise-related stress.

## 1. Introduction

The athletic horse has been selectively bred for centuries to optimize its innate running capabilities, resulting in animals with exceptional anatomical and functional adaptations for high-intensity exercise [1]. While genetically influenced, a horse’s performance is profoundly enhanced through systematic training. This process aims to increase aerobic capacity, muscle mass and vascularization, delay fatigue, and improve biomechanical efficiency and neuromuscular coordination [2,3].

However, strenuous physical exertion activates multiple physiological pathways, some of which can have adverse effects. The primary *loci minores resistentiae* in equine athletes include the musculoskeletal, respiratory, cardiovascular, and hematopoietic systems. Here, physiological disturbances, transient pathophysiological states, or even pathological dysfunctions often manifest, such as exercise-induced pulmonary hemorrhage, muscle soreness or myopathy, cardiac arrhythmias, and oxidative stress [4,5]. In response to such tissue damage—whether from inflammation, physical trauma, or extreme exertion—horses mount an acute-phase response (APR) [6,7,8,9,10,11,12]. This systemic reaction involves rapid metabolic and physiological changes, providing a non-specific, early defence mechanism until specific adaptive or immune responses are activated. The APR ultimately functions to increase host defense, minimize tissue damage, and accelerate recovery [6,7,13,14,15]. In the context of exercise, the APR is thought to be triggered by a combination of factors, including muscle microtrauma, transient hypoxia, and the release of damage-associated molecular patterns (DAMPs) from stressed tissues. This response serves to clear cellular debris, initiate tissue repair, and restore homeostasis. However, an excessively pronounced or prolonged APR can be detrimental, indicating a failure of recovery and potentially contributing to the pathogenesis of overtraining syndrome.

A cornerstone of the APR is the rapid hepatic synthesis and release of acute-phase proteins (APPs), whose plasma concentrations can increase several hundred-fold within hours of an inflammatory stimulus [6,13,16,17,18]. These proteins are classified as positive or negative based on their concentration changes. Their baseline levels show inter-individual, age, and sex-dependent variation, with significant differences also existing between species [7,18,19,20,21,22]. In the horse, serum amyloid A (SAA) and C-reactive protein (CRP) are considered major positive APPs capable of increasing 10- to 1000-fold during inflammation. Other proteins like haptoglobin (Hp) and ceruloplasmin (Cp) also participate, though their increase is typically more moderate (2- to 10-fold) and sustained, placing them in the category of moderate or minor APPs [19,23,24].

Serum Amyloid A (SAA), first isolated from equine serum during an APR, is an extremely sensitive marker [25]. SAA has a short half-life of approximately 24–48 h, and its concentration can peak within 48 h of an acute inflammatory stimulus [22]. Its concentration can rise 4 to 230 times above baseline within 48 h in conditions ranging from infections and surgery to systemic inflammation [18,22,26,27,28]. This wide dynamic range makes SAA particularly useful for detecting inflammation of varying severity. For instance, while a localized inflammatory process like sterile arthritis can induce a several-hundred-fold increase [28], the systemic stress from strenuous exercise has been reported to cause more moderate, though still significant, elevations, typically in the range of a 2- to 10-fold increase [10,11]. Factors such as the type, duration, and intensity of exercise, as well as the fitness level of the horse, are thought to influence the magnitude of this response. Beyond its role as a marker, SAA is functionally involved in chemotaxis, macrophage activation, lipid metabolism, and the regulation of inflammatory processes, including the induction of matrix-degrading enzymes [22,29,30,31]. During the APR, SAA becomes the principal apolipoprotein of high-density lipoprotein (HDL), displacing apoA-I and altering HDL’s function from anti-inflammatory to pro-inflammatory, a change critical for cholesterol redistribution to sites of tissue repair [32,33,34,35,36]. SAA can also be synthesized extrahepatically (e.g., inflamed joints), underscoring its local role in inflammation [37,38,39]. This local production can contribute to early and high concentrations of SAA in compartments like synovial fluid, peaking before the systemic response [38]. Due to its sensitivity and dynamic range, SAA is widely regarded as a valuable non-specific indicator of inflammation and general health status in equine [10,11,25,28,40,41,42,43,44,45,46,47].

Haptoglobin (Hp), with a short half-life of 2–4 days, is a sensitive marker of acute tissue damage [6,19,22,48]. Its synthesis is stimulated by cytokines such as IL-6. Hp’s primary function is to bind free hemoglobin released during intravascular hemolysis, thereby exerting antibacterial and antioxidant effects through iron sequestration. It also possesses anti-inflammatory properties, inhibiting prostaglandin synthesis and attenuating the leukocyte respiratory burst [6,19,48]. In exercising horses, Hp has been studied with variable results; some studies report a mild increase after intense exercise, while others show no change or even a decrease, possibly due to consumption from exercise-induced hemolysis [9,49].

Ceruloplasmin (Cp) is a copper-transporting glycoprotein and oxidase with a half-life of approximately four days [22,50]. It plays a crucial role in iron metabolism by oxidizing ferrous iron to its ferric form, enabling binding to transferrin. Cp contributes to antioxidant defense by binding copper and heme, and by scavenging reactive oxygen species [22,51]. Its synthesis increases during the APR, though typically later than that of SAA. As a moderate APP, its concentrations typically peak later in the inflammatory response, often between 72 and 96 h after the initial stimulus [22].

Studies monitoring APPs following inflammatory challenges, such as induced arthritis, show that SAA concentrations rise sharply within 16 h, peak by 36–48 h, and may take up to 15 days to normalize, while Hp increases more gradually [28]. These findings confirm that a localized insult can elicit a measurable systemic APR.

Despite established knowledge of the APR in disease, the impact of different *exercise paradigms* on this system in horses requires further elucidation. Exercise is a physiological stressor that can induce muscle microtrauma, hemolysis, and a systemic cytokine response, potentially activating the APR [9,10,11,52,53,54,55]. However, the effects of exercise *intensity* versus *duration* on the magnitude and kinetics of key APPs like SAA, Hp, and Cp is not well characterized. Such knowledge is vital for distinguishing physiological adaptation from excessive inflammation associated with overtraining or subclinical pathology.

For this investigation, we selected SAA, Hp, and Cp because they represent a spectrum of APP responses in the horse. SAA is the major, highly sensitive APP with a rapid and dramatic response. Hp is a moderate APP with a primary function in binding hemoglobin, making it potentially responsive to exercise-induced hemolysis. Cp is also a moderate APP, but with a key role in iron metabolism and antioxidant defense, linking it to the oxidative stress associated with prolonged exercise. This panel allows us to capture different facets of the potential inflammatory and physiological response to exercise—inflammation, hemolysis, and oxidative stress—providing a more comprehensive picture than a single marker like fibrinogen or CRP, whose responses in horses can be less specific or more variable.

Therefore, this study aimed to: (1) monitor and compare the post-exercise kinetics of serum amyloid A (SAA), haptoglobin (Hp), and ceruloplasmin (Cp) in Thoroughbred racehorses following two distinct exercise stimuli that differ fundamentally in their intensity and duration—a short, high-intensity gallop race (2400 m) and a long, low-intensity endurance race (40 km); and (2) correlate the intensity and duration of physical activity with the dynamics of these acute-phase reactants. We hypothesized that prolonged endurance exercise would provoke a more pronounced and sustained late-phase acute-phase response compared to brief, high-intensity gallop exercise.

## 2. Materials and Methods

### 2.1. Ethical Approval

All procedures involving animals were conducted in accordance with the European Union Directive 2010/63/EU and the national legislation on animal welfare for scientific purposes. The study protocol was reviewed and approved by the Ethics Committee of the Faculty of Veterinary Medicine, University of Belgrade (Approval No. 323-07-10294/2021-05). The owners of all horses provided informed consent for participation in the study.

### 2.2. Animals and Study Design

The study was designed as a prospective, observational cohort investigation conducted during the competition season (May–June 2020). Two groups of clinically healthy, trained male Thoroughbred racehorses (a mix of stallions and geldings) were included.

Group I (Gallop): This group consisted of 12 horses, aged 3–5 years (mean ± SD: 4.1 ± 0.8 years), which had successfully completed an official 2400-m flat gallop race on a sand track, finishing in 1st, 2nd, or 3rd place. The average race time was 2 min 42 s ± 5 s.

Group II (Endurance): This group comprised 13 horses, aged 3–8 years (mean ± SD: 6.2 ± 1.5 years), which completed an official 40-km endurance race in accordance with International Equestrian Federation (FEI) regulations. The average ride time was 2 h 15 min ± 18 min, and all horses passed the mandatory veterinary checks at the finish line.

All horses were of the Thoroughbred breed. The endurance horses were competing in a 40 km race, a distance at which Thoroughbreds sometimes compete. All horses had been in active training for their respective disciplines for at least two years prior to the study. The gallop horses were in training for flat racing, typically involving high-speed work 2–3 times per week. The endurance horses were conditioned for long-distance events, with training consisting of regular, prolonged trotting and cantering sessions. All horses were at a similar, high level of fitness appropriate for their discipline at the time of the study, as judged by their trainers and the study veterinarians.

All horses underwent a standard clinical examination before inclusion to confirm the absence of fever, lameness, or systemic illness. Their routine training and nutritional regimens were maintained throughout the study period.

The gallop races took place on a single race day, while the endurance race occurred on a separate day two weeks later. All 13 horses in Group II competed in the same 40-km endurance race on the same day. All sampling for each respective group was timed accordingly.

### 2.3. Experimental Procedures and Sample Collection

Blood samples were collected via jugular venipuncture using sterile vacuum tubes without anticoagulant (Vacuette^®^ Serum Clot Activator, Greiner Bio-One GmbH, Kremsmünster, Austria).

For Group I (Gallop), sampling was performed at three time points:

o T0: 24 h before the race.
o T1: 72 h post-race.
o T2: 96 h post-race.

For Group II (Endurance), sampling was performed at seven time points:

o T0: 24 h before the race.
o T1: Immediately after finishing the race (<10 min post-completion).
o T2: 48 h post-race.
o T3: 72 h post-race.
o T4: 96 h post-race.
o T5: 120 h post-race.
o T6: 144 h post-race.

For Group I (Gallop), post-exercise sampling was performed at 72 and 96 h. The monitoring period therefore extended to 96 h after the race.

The sampling schedule for this group was designed based on previous reports indicating that short, high-intensity racing exercise in Thoroughbreds typically induces either no significant increase or only a modest and transient elevation of SAA, often resolving within 24–72 h [6,11]. In contrast, moderate acute-phase proteins such as ceruloplasmin and haptoglobin are known to peak later in the inflammatory cascade, generally between 72 and 96 h following a stimulus [18,23].

Therefore, sampling at 72 and 96 h was selected to capture potential delayed peaks of moderate acute-phase proteins while minimizing unnecessary repeated venipuncture under field race conditions.

Samples were allowed to clot at room temperature for 30 min and then centrifuged at 3000× *g* for 10 min. The collected serum was aliquoted and stored at −80 °C until analysis to prevent protein degradation.

### 2.4. Biochemical Analyses

Analyses were performed in a single batch for each analyte to minimize inter-assay variation.

Serum Amyloid A (SAA): Concentration was determined using a commercially available, equine-specific sandwich ELISA kit (Tridelta Phase™ Range SAA EIA Kit, Tridelta Development Ltd., Wicklow, Ireland; Catalog #TP-802). The assay was performed strictly according to the manufacturer’s instructions. The absorbance was read at 450 nm (with a 630 nm reference) using a microplate reader (Mod. A1, Nubenco Enterprises Inc., Paramus, NJ, USA). The reported detection range of the kit is 0.5–200 µg/mL, with intra- and inter-assay coefficients of variation (CV) of <8%. Results are expressed in mg/L.

Haptoglobin (Hp): Concentration was determined using a commercially available, equine-specific colorimetric assay (Tridelta Phase™ Haptoglobin Assay, Tridelta Development Ltd., Wicklow, Ireland; Catalog #TP-801) based on the preservation of peroxidase activity of the hemoglobin-haptoglobin complex. The procedure followed the manufacturer’s protocol. Absorbance was read at 630 nm. The assay’s detection range is 0.05–2.5 mg/mL, with intra- and inter-assay CVs of <7%. Results are expressed in mg/mL.

Ceruloplasmin (Cp): Ferroxidase activity, proportional to Cp concentration, was measured spectrophotometrically using *p*-phenylenediamine dihydrochloride as the substrate, according to the method of Sunderman and Nomoto [56]. Briefly, serum was incubated with acetate buffer (pH 5.5) and substrate. The rate of formation of the purple oxidation product was measured by the increase in absorbance at 530 nm over 30 min at 37 °C using a Cecil Aurius CE 2021 UV/VIS spectrophotometer (Cecil Instruments Ltd., Cambridge, UK). Cp concentration was calculated using a molar extinction coefficient and expressed in mg/L. The intra-assay CV for this method in our laboratory was <5%.

### 2.5. Statistical Analysis

An a priori sample size calculation was performed using G*Power software (version 3.1.9.6). Based on pilot data for SAA variability and desiring to detect a moderate effect size (f = 0.25) with 80% power and an alpha of 0.05 in a repeated-measures design, a minimum of 10 horses per group was required. We enrolled 12 and 13 horses to account for potential dropouts.

Data were tested for normality using the Shapiro–Wilk test and are presented as mean ± standard deviation (SD). Because some APP data were not normally distributed and the study employed a repeated-measures design, statistical analysis was performed using linear mixed-effects models to conduct a two-way repeated-measures ANOVA, with Group (gallop vs. endurance) and Time (common time points: pre-exercise, 72 h, 96 h) as fixed factors, Horse ID as a random intercept, and the Group × Time interaction term. Time points with entirely missing data for one group (immediately after and 48 h after the gallop exercise) were excluded from the model. Denominator degrees of freedom were estimated using the Kenward–Roger method. When the Group × Time interaction was significant, results were summarized by the interaction and post-hoc comparisons of time points within each group versus the respective pre-exercise baseline (Dunnett-adjusted); significant differences are indicated in Table 1 using letters (*p* < 0.05). The relationship between APP concentrations at peak response time was assessed using Pearson’s or Spearman’s correlation coefficient (r).

Graphical presentation and statistical analysis were performed using GraphPad Prism software (Version 9.0 for Windows, GraphPad Software, San Diego, CA, USA) and R version 4.2.1 (lme4, lmerTest packages). A two-tailed *p*-value of <0.05 was considered statistically significant.

## 3. Results

**Figure 1:**
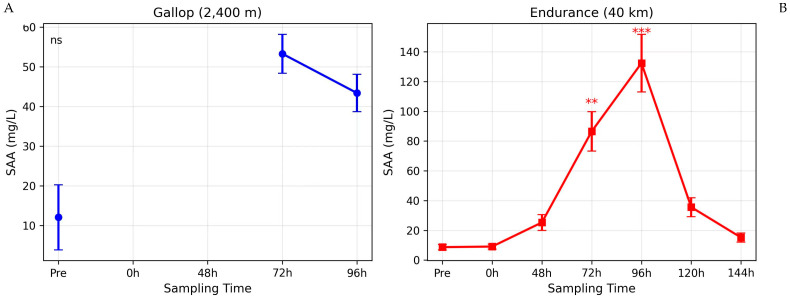
Serum amyloid A (SAA) kinetics after gallop and endurance exercise. (**A**) SAA concentrations in gallop horses (*n* = 12) at pre-exercise, 72 h, and 96 h post-race. (**B**) SAA concentrations in endurance horses (*n* = 13) at pre-exercise, immediately post-race, and at 48, 72, 96, 120, and 144 h post-race. Data are mean ± SD. ** *p* < 0.01, *** *p* < 0.001 *versus* pre-exercise (Dunnett’s test).

**Figure 2:**
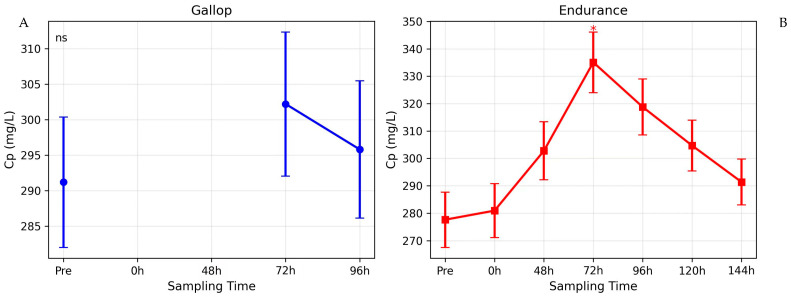
Ceruloplasmin (Cp) kinetics after gallop and endurance exercise. (**A**) Cp concentrations in gallop horses. (**B**) Cp concentrations in endurance horses. Data are mean ± SD. * *p* < 0.05 *versus* pre-exercise.

**Figure 3:**
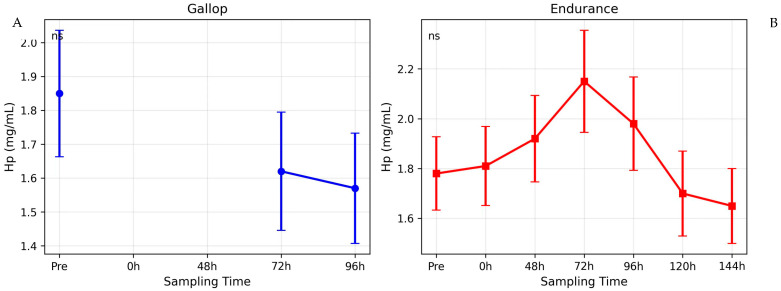
Haptoglobin (Hp) kinetics after gallop and endurance exercise. (**A**) Hp concentrations in gallop horses. (**B**) Hp concentrations in endurance horses. Data are mean ± SD. No significant changes were detected.

Descriptive statistics across all sampled time points are shown in Figure 1, Figure 2 and Figure 3; mixed-model results for the common time points (pre, 72 h, 96 h) are summarized in Table 1.

### 3.1. Serum Amyloid A (SAA)

The two-way mixed ANOVA revealed a highly significant Group × Time interaction for SAA (F_2_,_46_ = 8.76, *p* < 0.001), indicating that the temporal pattern of SAA differed between the gallop and endurance groups (Figure 1). Post-hoc comparisons showed an increase from pre-exercise at 72 h and 96 h in the endurance group, whereas no significant change from baseline was detected in the gallop group (Table 1).

### 3.2. Ceruloplasmin (Cp)

For Cp, the mixed ANOVA showed a significant Group × Time interaction (F_2_,_46_ = 3.65, *p* = 0.034), indicating that the two groups responded differently over time (Figure 2). Post-hoc comparisons identified a significant increase from pre-exercise at 72 h in the endurance group, while Cp did not change significantly over time in the gallop group (Table 1).

In Group I (Gallop), Cp concentrations remained stable throughout the observation period, with no significant differences between pre-exercise levels (286.8 ± 30.5 mg/L) and the 72 h or 96 h post-exercise measurements (*p* > 0.05).

Between-group comparisons at 72 h and 96 h showed no statistically significant differences (*p* = 0.102 and *p* = 0.187, respectively), likely due to inter-individual variability.

### 3.3. Haptoglobin (Hp)

For Hp, the mixed ANOVA revealed no significant Group × Time interaction (F_2_,_46_ = 0.59, *p* = 0.558) (Figure 3), and no post-exercise time point differed significantly from the respective pre-exercise baseline in either group (Table 1).

In Group I (Gallop), Hp concentrations displayed a slight, non-significant decreasing tendency from a pre-exercise mean of 1.81 ± 0.60 mg/mL to 1.56 ± 0.59 mg/mL at 96 h post-exercise (*p* > 0.05).

### 3.4. Between-Group Comparison at Common Time Points

**Figure 4:**
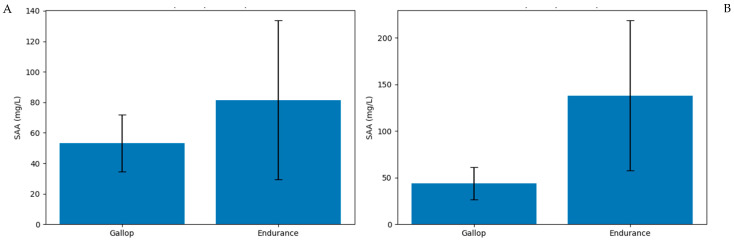
Between-group comparison of Serum amyloid A (SAA) concentrations at shared post-exercise time points. (**A**) 72 h post-exercise (*p* = 0.074). (**B**) 96 h post-exercise (*p* = 0.018). Bars represent mean ± SD.

Direct comparison of SAA concentrations between the gallop and endurance groups was performed at the shared post-exercise time points (72 h and 96 h). While SAA values did not differ significantly at 72 h (*p* = 0.074), concentrations were significantly higher in the endurance group at 96 h (*p* = 0.018) (Figure 4).

### 3.5. Correlation Analysis

No statistically significant correlations were identified between peak concentrations of SAA, Cp, and Hp within either group (all |r| < 0.40, *p* > 0.05). These findings suggest that each acute-phase protein may reflect partially distinct physiological pathways in response to exercise-induced stress.

## 4. Discussion

### 4.1. Overview of Main Findings

This study evaluated the acute-phase protein response in Thoroughbred racehorses subjected to two distinct exercise paradigms differing in intensity and duration. The principal finding was that prolonged endurance exercise induced a statistically significant and biologically substantial late-phase acute-phase response, as evidenced by marked increases in serum amyloid A (SAA) and moderate elevation in ceruloplasmin (Cp), whereas short, high-intensity gallop exercise did not elicit statistically significant changes in the measured acute-phase proteins at the evaluated time points.

### 4.2. Serum Amyloid A and the Late-Phase Response to Exercise

The marked increase in SAA concentrations following the endurance race reinforces its classification as a major acute-phase protein and a highly sensitive biomarker of systemic inflammation in horses [18,21,25,28]. The approximately 15-fold elevation at 96 h post-exercise, although markedly lower than increases observed during infectious or surgical inflammation, reflects a moderate but biologically meaningful systemic response consistent with exercise-induced sterile inflammation [25,32,34].

The delayed peak observed at 72–96 h differs from the rapid increase in SAA typically seen in acute infectious patterns, where concentrations often peak within 24–48 h. [28,40]. This delayed pattern suggests that the inflammatory stimulus associated with prolonged exercise may be cumulative rather than acute. Repeated muscle microtrauma, sustained metabolic strain, oxidative stress, and cytokine signaling likely contribute to ongoing hepatic stimulation of SAA synthesis during recovery rather than immediately during exertion [52,53,54,55].

In contrast, the gallop group demonstrated a non-significant trend toward increased SAA at 72 and 96 h. Although the absence of statistical significance suggests a limited systemic response at these time points, it must be acknowledged that early post-exercise sampling (immediate and 24–48 h) was not performed in this group. Previous studies indicate that short, high-intensity racing may induce modest and transient SAA elevations that normalize within 24–72 h [10,11]. Therefore, we cannot exclude the possibility that an earlier SAA peak occurred and resolved prior to our first post-exercise sampling at 72 h.

Nevertheless, the substantial difference observed at 96 h—with a large effect size and confidence interval excluding zero—indicates that the endurance modality produced a more persistent systemic inflammatory activation compared to gallop exercise.

Importantly, the significant Group × Time interaction for SAA confirms that the pattern of change differed between exercise modalities at the measured time points, supporting the conclusion that exercise duration contributes substantially to late-phase systemic inflammatory activation.

### 4.3. Ceruloplasmin Dynamics and Oxidative Stress Adaptation

Ceruloplasmin concentrations increased significantly 72 h after endurance exercise, consistent with its classification as a moderate and later-rising acute-phase protein [51]. Although between-group differences did not reach statistical significance, the effect size at 72 h was moderate-to-large (d = 0.96), suggesting a biologically relevant trend that may have been underpowered due to sample size and interindividual variability.

Ceruloplasmin plays a crucial role in iron metabolism and antioxidant defense [22,50]. Prolonged exercise is associated with increased reactive oxygen species production and potential subclinical hemolysis, both of which may stimulate Cp synthesis. The delayed Cp response aligns with its slower kinetic profile relative to SAA and suggests a secondary phase of adaptive antioxidant regulation rather than immediate inflammatory activation [35,50,57].

The absence of a significant Cp response in the gallop group further supports the interpretation that exercise duration, rather than peak intensity alone, is the principal determinant of sustained systemic inflammatory signaling.

### 4.4. Haptoglobin and the Absence of a Marked Hemolytic Signal

Haptoglobin concentrations did not change significantly following either exercise modality. While the endurance group exhibited a mild increasing tendency at 72 h and the gallop group showed a slight non-significant decrease, these changes were modest and of limited magnitude.

The small between-group effect size observed for Hp suggests that exercise-induced hemolysis, if present, was insufficient to provoke a pronounced hepatic acute-phase response. This finding aligns with previous literature reporting variable or minimal Hp response following strenuous exercise in horses [1,9,58]. The slight, non-significant decrease observed after gallop exercise may be attributable to exercise-induced intravascular hemolysis, a well-recognized phenomenon in high-speed work, leading to transient Hp consumption [59].

Collectively, the differential responses of SAA, Cp, and Hp highlight that not all acute-phase proteins are equally responsive to physiological exercise stress and that SAA remains the most sensitive marker for detecting cumulative systemic activation.

### 4.5. Magnitude-Based Interpretation and Clinical Implications

A strength of the present study lies in the integration of effect size and confidence interval reporting alongside *p*-values. While statistical significance provides information about probability, effect size offers insight into biological relevance. The large effect size observed for SAA at 96 h supports the interpretation that endurance exercise induces a clinically meaningful systemic response, even within a relatively small sample size.

From a practical standpoint, monitoring SAA concentrations in the recovery period following prolonged exercise may provide valuable information regarding systemic stress and recovery adequacy. A moderate and transient elevation likely reflects physiological adaptation and tissue repair processes. A lack of increase may indicate that the effort did not represent a substantial physiological challenge, or that the horse recovered rapidly due to a high fitness level. Conversely, excessive or prolonged elevation beyond expected recovery windows (e.g., SAA > 200 mg/L for >5 days) may signal insufficient recovery, overtraining, or underlying pathology.

Given the substantial interindividual variability observed, establishing individual baseline values and longitudinal monitoring within the same horse may be more informative than relying solely on population-based reference intervals.

### 4.6. Study Limitations

Several limitations must be acknowledged.

First, the study design involved two independent groups rather than a crossover model, limiting control over individual variability.

Second, although sample size met a priori power calculations for moderate effects, subtle differences—particularly for moderate acute-phase proteins—may have remained undetected.

Third, the absence of early post-exercise sampling (immediate and 24–48 h) in the gallop group restricts direct comparison of early acute-phase kinetics between exercise types. It is therefore possible that a transient SAA elevation occurred in the gallop horses prior to 72 h and resolved before sampling. Consequently, our conclusions primarily reflect differences in the magnitude and persistence of the late-phase response rather than the complete temporal kinetics of acute-phase activation.

Fourth, cytokine concentrations and oxidative stress biomarkers were not measured, limiting mechanistic insight into the pathways linking exercise modality and hepatic acute-phase synthesis.

Finally, while restricting the study to Thoroughbreds reduced breed-related variability, this also limits generalizability to breeds specifically selected for endurance performance, such as Arabians, which may exhibit different inflammatory kinetics. Future studies integrating acute-phase proteins with inflammatory and metabolic biomarkers would further clarify the pathways linking exercise and systemic inflammation.

## 5. Conclusions

This study demonstrates that prolonged endurance exercise induces a measurable and biologically substantial late-phase acute-phase response in Thoroughbred racehorses, characterized by significant increases in serum amyloid A (SAA) and moderate elevations in ceruloplasmin (Cp). In contrast, short, high-intensity gallop exercise did not elicit statistically significant changes in the evaluated acute-phase proteins at the measured time points.

The significant Group × Time interaction observed for SAA, together with the large effect size at 96 h post-exercise, indicates that exercise duration plays a decisive role in modulating the persistence and magnitude of systemic inflammatory activation. These findings support the concept that cumulative physiological stress associated with prolonged effort, rather than peak intensity alone, is the primary driver of sustained acute-phase protein synthesis.

Serum amyloid A emerged as the most sensitive biomarker of exercise-induced systemic stress, displaying both statistically significant and biologically meaningful changes following endurance exercise. Monitoring SAA concentrations during recovery may therefore provide a practical tool for assessing post-exercise inflammatory load, guiding training management, and identifying horses at risk of inadequate recovery.

However, because early post-exercise sampling was not performed in the gallop group, the present conclusions primarily reflect differences in the late-phase response (72–96 h) rather than the complete temporal kinetics of acute-phase activation. Future studies incorporating early and continuous post-exercise sampling, as well as inflammatory cytokine and oxidative stress markers, would further elucidate the mechanistic pathways linking exercise modality to systemic inflammatory regulation.

Overall, the present findings contribute to a more nuanced understanding of exercise-induced sterile inflammation in equine athletes and reinforce the importance of considering both the magnitude and duration of physiological stress when evaluating recovery and performance.

## Figures and Tables

**Table 1:** Serum amyloid A (SAA), ceruloplasmin (Cp), and haptoglobin (Hp) concentrations in Thoroughbred racehorses subjected to a 2400 m gallop race (Group I, *n* = 12) or a 40 km endurance race (Group II, *n* = 13) at the common sampling time points used in the mixed-model analysis (pre-exercise, 72 h, 96 h). Different letters within a group indicate significant differences *versus* pre-exercise (Dunnett-adjusted, *p* < 0.05). The rightmost column reports the Group × Time interaction *p*-value from the mixed model.

Parameter	Time Point	Gallop (*n* = 12)	Endurance (*n* = 13)	Group × Time *p*
SAA (mg/L)	Pre	12.0 ± 26.7 a	9.0 ± 7.1 a	<0.001
	72 h	53.1 ± 18.7 a	81.5 ± 52.1 b	
	96 h	43.8 ± 17.2 a	138.2 ± 80.4 b	
Cp (mg/L)	Pre	286.8 ± 30.5 a	272.5 ± 33.1 a	0.034
	72 h	298.5 ± 32.1 a	333.5 ± 40.2 b	
	96 h	291.2 ± 29.8 a	317.2 ± 38.7 a	
Hp (mg/mL)	Pre	1.81 ± 0.60 a	1.78 ± 0.49 a	0.558
	72 h	1.62 ± 0.55 a	2.18 ± 0.68 a	
	96 h	1.56 ± 0.59 a	2.01 ± 0.62 a	

## Data Availability

All data generated or analysed during this study are included in this published article. The datasets used and/or analysed in this study are available from the corresponding author upon reasonable request.

## Abbreviations

The following abbreviations are used in this manuscript:

APR	Acute-Phase Response
APPs	Acute-Phase Proteins
SAA	Serum Amyloid A
CRP	C-Reactive Protein
Hp	Haptoglobin
Cp	Ceruloplasmin
HDL	High-Density Lipoprotein
FEI	International Equestrian Federation
CV	Coefficients of Variation
SD	Standard Deviation
